# STARTING A TESTOSTERONE AND EXERCISE PROGRAM AFTER HIP INJURY (STEP-HI) TRIAL: RATIONALE, METHODS, AND FIDELITY

**DOI:** 10.1093/geroni/igz038.1568

**Published:** 2019-11-08

**Authors:** Ellen Binder

**Affiliations:** Washington University School of Medicine, St. Louis, Missouri, United States

## Abstract

The STEP-HI study is an ongoing multi-center, randomized, controlled, double-blinded clinical trial that is evaluating whether six months of topical testosterone therapy combined with a supervised center-based exercise-training program can improve mobility, functional performance, and quality of life after hip fracture, compared to exercise training alone or Enhanced Usual Care. Female hip fracture patients ≥ 65 yrs. old who are living in the community or assisted living are being randomized within 16 weeks of surgical repair for the fracture, and re-evaluated 24 weeks later. Three hundred patients will be recruited from six clinical centers in the USA. This presentation discusses the rationale and study design, including innovative methods for fidelity monitoring of exercise procedures across the clinical sites. Strategies for recruitment and related challenges will be discussed. Preliminary enrollment and fidelity data will be presented.

